# The impact of Sars-Cov-2 infection on the wound healing of cervical treatment in patients with squamous intraepithelial lesions: a retrospective cohort study

**DOI:** 10.3389/fmed.2023.1222767

**Published:** 2023-12-07

**Authors:** Lili Xu, Yuying Wu, Chengzhi Li, Renfeng Zhao, Zhibiao Wang

**Affiliations:** ^1^State Key Laboratory of Ultrasound in Medicine and Engineering, College of Biomedical Engineering, Chongqing Medical University, Chongqing, China; ^2^Chongqing Key Laboratory of Biomedical Engineering, Chongqing Medical University, Chongqing, China; ^3^Department of Gynecology, The People's Hospital of Guangxi Zhuang Autonomous Region, Guangxi Academy of Medical Sciences, Nanning, China

**Keywords:** cervical squamous intraepithelial lesion, loop electrosurgical excision procedure, ablative treatment, SARS-CoV-2, wound healing

## Abstract

**Objective:**

SARS-CoV-2 infection has been associated with an increase in inflammatory factors, a weakening of the immune system, and a potentially delay in wound healing following surgery or ablative treatment. In this retrospective cohort study, we aimed to investigate the impact of SARS-CoV-2 infection on wound healing following cervical treatment in patients with squamous intraepithelial lesions (SIL).

**Method:**

From November 2022 to February 2023, patients with SIL who underwent cervical ablative treatment or loop electrosurgical excision procedure at the People’s Hospital of Guangxi Zhuang Autonomous Region, China, were enrolled in the study. Of these, 29 patients who developed symptoms of SARS-CoV-2 infection and confirmed by an antigen test within one month after cervical treatment were included as experimental group, while the other 31 patients who received cervical treatment after recovering from SARS-CoV-2 infection were included in the control group. The cervical wound condition of all patients was documented using colposcopy immediately and one month after the procedure. Image J software was utilized to analyze the wound healing rate at one month post-treatment, and the wound healing status between two groups was compared. A vaginal discharge examination was performed before and one month after cervical treatment.

**Results:**

No significant differences in age, severity, treatment, or time between groups. Experimental group had significantly lower healing rate 83.77(62.04, 97.09) % than control 98.64(97.10, 99.46)%，*p* < 0.001, and a higher scab non-shedding rate (24.14% vs. 3.22%, *p* = 0.024). Among patients who were infected with SARS-CoV-2 after undergoing cervical treatment, we observed 5 out of 7 patients (71.43%) contracted SARS-CoV-2 within 2 weeks after cervical treatment. No significant correlation was found between white blood cell count or leukocyte esterase in vaginal discharge and delayed wound healing of the cervix (*p* = 0.947 and 0.970, respectively).

**Conclusion:**

SARS-CoV-2 infection may prolong the healing time of cervical treatment in patients with SIL. To minimize the risk of delayed healing, it’s crucial for patients to avoid viral infections such as SARS-CoV-2 within the first month of treatment. Taking necessary precautions to prevent infection is essential for successful cervical treatment outcomes in patients with SIL.

## 1 Introduction

Cervical squamous intraepithelial lesion (SIL) is a condition characterized by abnormal changes in cervical squamous cells (1). Although most low-grade squamous intraepithelial lesions (LSILs) can regress naturally within 1–2 years, while (HSILs) have a higher potential for malignant transformation (2). Current treatment high-grade squamous intraepithelial lesionsmethods for SIL comprise ablative treatments (such as cryotherapy, radiofrequency, and focused ultrasound treatments) and excision procedures (including cold knife conization, cervical loop electrosurgical excision procedure, and laser conization) (3–5). In the past few years, there has been an increase in the occurrence of SIL among younger woman (6). As a result, clinicians and patients are not only concerned about lesion clearance, but also paying attention to cervical wound healing, which has emerged as a new area of interest.

SARS-CoV-2, a novel coronavirus, emerged in 2019 and caused a global pandemic of acute respiratory illness named SARS-CoV-2 (7–9). With the deepening of research, it has been found that SARS-CoV-2 not only affects the respiratory system, but also the digestive system, nervous system, cardiovascular system, endocrine system, reproductive system, and can cause a decrease in the body’s immunity, leading to secondary bacterial infections (10–15). However, little is known about the impact of SARS-CoV-2 infection on cervical wound healing after treatment. In this study, we investigated the wound healing status of patients who were infected with SARS-CoV-2 within one month after cervical treatment and compared them with a control group of patients who underwent cervical treatment after the disappearance of infection symptoms. The findings are reported as follows:

## 2 Materials and methods

### 2.1 Study population

A total of 60 patients, aged 19 to 53 years old, who underwent cervical treatment for squamous intraepithelial lesions (SIL) at the gynecology cervical clinic of the People’s Hospital of Guangxi Zhuang Autonomous Region from November 2022 to February 2023 were recruited as the study population. Informed consent was obtained from all subjects involved in the study. The inclusion criteria for patients were a diagnosis of SIL using the three-step method of cervical cytology, which including HPV test, colposcopy, and cervical biopsy.

The exclusion criteria were including: patients with acute or subacute genital infections, genital malignancy, a history of hysterectomy or pelvic radiotherapy, a history of cervical excision, cervical ablation, or medication treatment, pregnant or lactating women, uncontrolled diabetes, hyperthyroidism or hypothyroidism, severe cardiovascular, cerebral, pulmonary, hepatic, or renal dysfunction, or comorbidities of immunological disorders or immunosuppressive drug use. The study flowchart is attached in the Supplementary materials (Supplementary material 1).

The experimental group consisted of 29 patients who exhibited symptoms (such as fever, sore throat, and cough) and were confirmed to have SARS-CoV-2 infection through antigen testing within one month of receiving cervical treatment. The control group comprised t31 patients who underwent cervical treatment at least one week after their SARS-CoV-2 symptoms had resolved. The study was conducted in accordance with the Declaration of Helsinki and was approved by the Ethics Committee of Guangxi Zhuang Autonomous Region People’s Hospital (no. KY-KJT02023-10) on August 01, 2022.

### 2.2 Treatment methods

All cervical treatments were performed 3–7 days after the end of menstruation, excluding patients who have reached menopause. To assess the extent of the cervical lesion, acetic acid and iodine are applied to stain the junction of the cervical squamous epithelium and columnar epithelium. Local anesthesia with 1% lidocaine is administered at the 4 quadrants of the cervix. The cervical squamous intraepithelial lesions are treated using either loop electrosurgical excision procedure (LEEP) or ablative treatment such as focused ultrasound or radiofrequency ablation. Details regarding the indications for patients undergoing LEEP excision or ablative treatment can be found in the Supplementary materials (Supplementary material 2).

The loop electrosurgical excision procedure (LEEP) is performed using a triangular-shaped electrosurgical knife with a length of 15–20 mm, The treatment area is set to approximately 5 mm from the outer edge of the lesion, utilizing a cutting-coagulation mode with a power setting of 40 W. Depending on the type of transformation zone, different lengths of lesion tissue are removed (7–10 mm for type 1, 10–15 mm for type 2, and 15–25 mm for type 3). Following excision, a ball-shaped electrode is used to perform electrocoagulation for hemostasis at the surgical site.

The power setting for focused ultrasound therapy is between 3.5 and 4.5 W. Treatment is performed in a circular scanning pattern from the lesion area toward the normal area. During the treatment, the focused ultrasound probe is kept in close contact with the treatment area. The treatment should be stopped when local tissues become concave or hardened. The treatment range should extend beyond the edge of the cervical lesion by approximately 2–5 mm. The power for radiofrequency ablation is set to 30 W. The treatment area extends beyond the area that tests positive in acetic acid and iodine tests by 2–5 mm. An auto-coagulator knife is used to ablate the cervical epithelium from the inside out, until the epithelium is thermocoagulated to a light yellow color and the wound has formed a shallow cone shape (16).

### 2.3 Cervical wound healing evaluation after treatment

Assessing the rate of wound healing is a key factor in evaluating the progress of wound recovery. A comprehensive evaluation of the healing process includes recording the cervical wound condition immediately after treatment and at a specified time-point after treatment. The wound area is measured using Imagine J software (National Institutes of Health, Bethesda, MD). To calculate the wound healing rate, the formula [(treatment wound area - remaining wound area)/treatment wound area] × 100% is applied. This approach enables clinicians to quantitatively assess the healing process and monitor the progress of wound closure over time. Specifically, the calculation is performed on the 30th day after treatment, providing a comprehensive evaluation of healing rate.

### 2.4 Vaginal discharge test before and one month after treatment

Before undergoing cervical treatment, each patient underwent a vaginal discharge examination to rule out vaginal inflammation. During the one-month follow-up vaginal colposcopy after receiving cervical treatment, another examination of vaginal discharge was conducted to assess the vaginal microbiota and inflammatory condition.

## 3 Statistical analysis

Quantitative variables were expressed as mean and standard deviation (SD) or median (25th percentile, 75th percentile) for non-normally distributed quantitative variables. The comparison of wound healing rates between two groups is conducted with Wilcoxon rank sum test. The proportion of patients with non-shedding scabs within 30 days after treatment in the two groups was analyzed using the chi-square test. Chi-square test for contingency table was used for investigate the relationship between white blood cell count or leukocyte esterase of vaginal discharge and delayed wound healing following cervical treatment. All statistical analyses were performed using SPSS software 27.0 (SPSS Inc., Chicago, IL, United States). A *p* value <0.05 was considered statistically significant.

## 4 Results

There were no significant differences between the two groups in terms of age, disease severity, treatment methods, or treatment duration, as indicated in Table 1. The mean time of SARS-CoV-2 infection in post-treatment infection group is 15.83 ± 9.74 days after cervical treatment, and the mean time of SARS-CoV-2 infection in treatment after infection recovered group is 39.13 ± 9.80 days before cervical treatment.

**Table 1:** Baseline demographic and clinical characteristics in the experimental group (patients with SARS-CoV-2 infection after cervical treatment) and control group.

Indices	Experimental group (*n* = 29)	Control group (n = 31)	*F* or *X^2^* value	*p*-value
Age(years)	34.00 ± 6.47	35.58 ± 6.64	0.111	0.740
LSIL	19	24	1.045	0.307
HISL	10	7		
Treatment method				
LEEP	10	12	0.115	0.734
Ablative treatment	19	19		

Baseline characteristics in the experimental group and control group. There were no statistically significant differences in baseline characteristics, severity of cervical lesions, or treatment received between the experimental and control groups.

LSIL, low grade squamous intraepithelial lesions; HSIL, high grade squamous intraepithelial lesions; LEEP, loop electrosurgical excision procedure; Ablative treatment, including focused ultrasound ablation and radiofrequency ablation.

**Figure 1:**
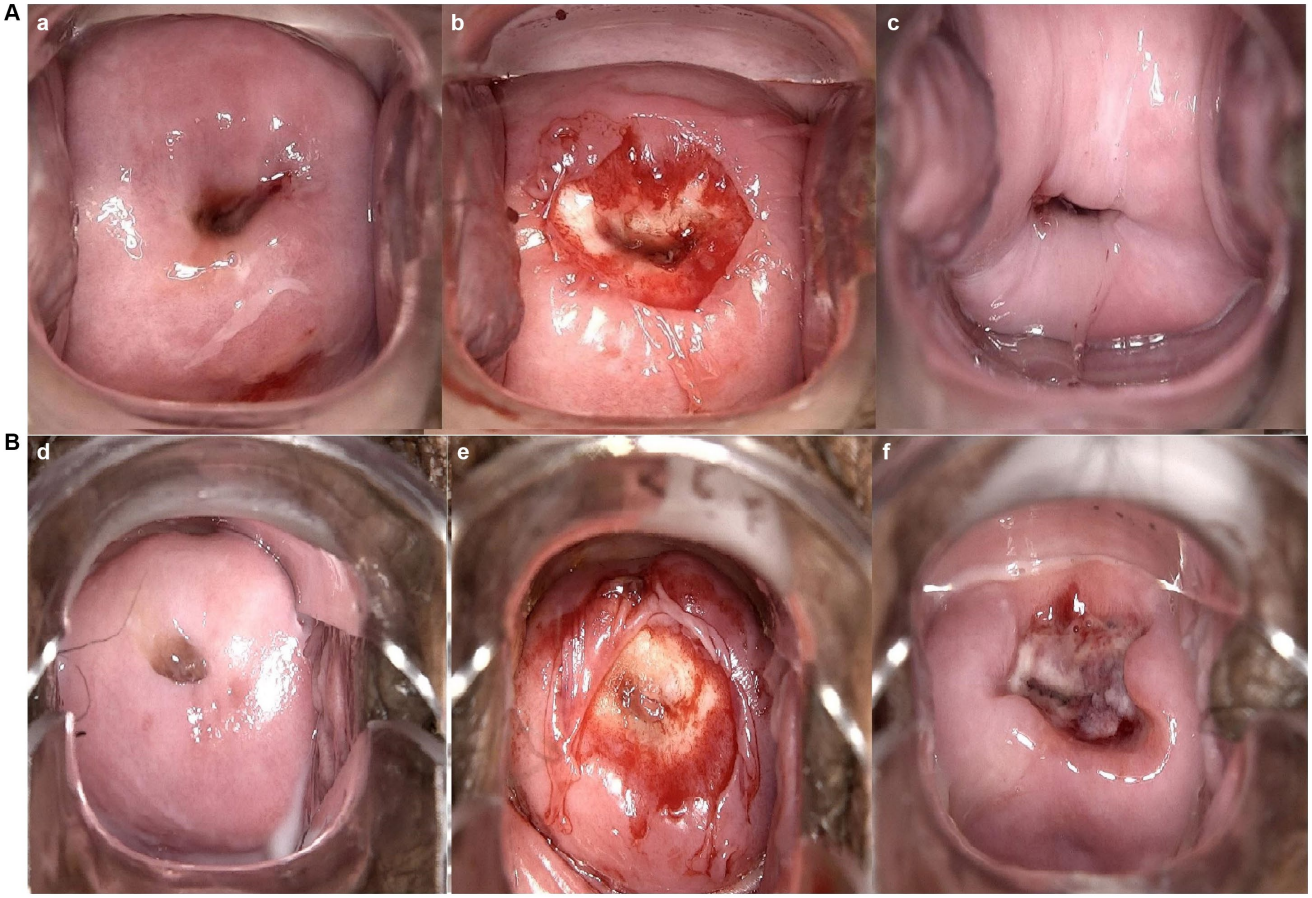
Vaginal colposcopy images of patients who received focused ultrasound ablation for LSIL in different groups. (a,d) are vaginal colposcopy images before treatment; (b,e) are vaginal colposcopy images taken immediately after the procedure; (c), are vaginal colposcopy images taken one month after the cervical treatment. **(A)** Shows vaginal colposcopy images of a patient in control group. Which Indicates good healing of the cervical wound after treatment. **(B)** Shows vaginal colposcopy images of a patient in experimental group. Which shows that the patient’s cervical wound was still unhealed one month after the cervical treatment.

**Figure 2:**
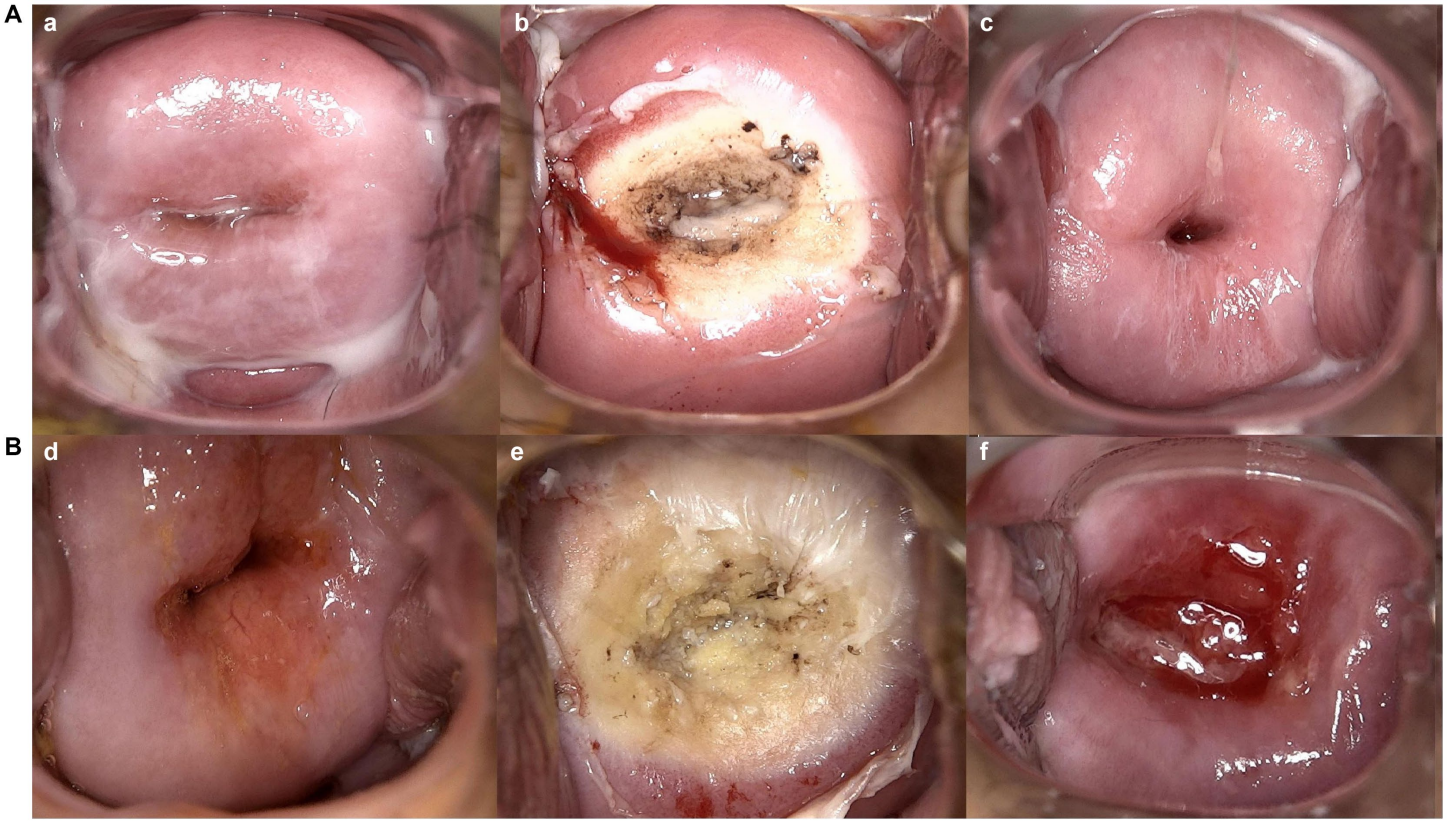
Vaginal colposcopy images of patients who received radiofrequency ablation for LSIL in different groups. (a, d) are vaginal colposcopy images before treatment; (b, e) are vaginal colposcopy images taken immediately after the procedure; (c), are vaginal colposcopy images taken one month after the cervical treatment. **(A)** shows vaginal colposcopy images of a patient in control group who received radiofrequency ablation for SIL 26 days after SARS-CoV-2 infection. Which Indicates good healing of the cervical wound after treatment. **(B)** Shows vaginal colposcopy images of a patient in experimental group who infected SARS-CoV-2 10 days after radiofrequency ablation. Which shows that the patient’s cervical wound was still unhealed one month after the cervical treatment.

**Figure 3:**
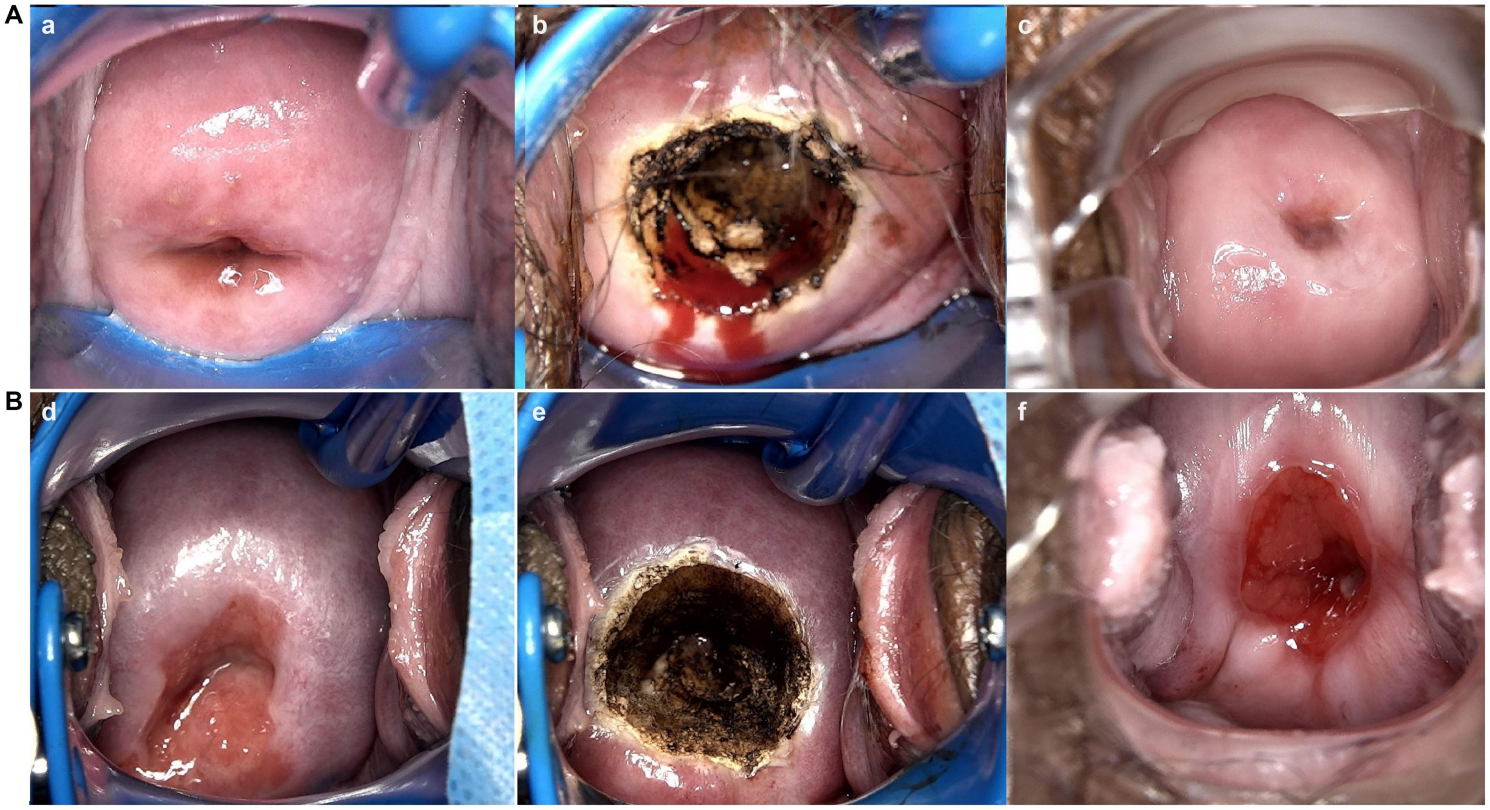
Vaginal colposcopy images of patients who received LEEP treatment for HSIL in different groups. (a,d) are vaginal colposcopy images before treatment; (b,e) are vaginal colposcopy images taken immediately after the procedure; (c), are vaginal colposcopy images taken one month after the cervical treatment. **(A)** Shows vaginal colposcopy images of a patient in control group who received LEEP excision for HSIL 38 days after SARS-CoV-2 infection. Which Indicates good healing of the cervical wound after treatment. **(B)** Shows vaginal colposcopy images of a patient in experimental group who infected SARS-CoV-2 16 days after LEEP excision. Which shows that the patient’s cervical wound was still unhealed one month after the cervical treatment.

**Figure 4:**
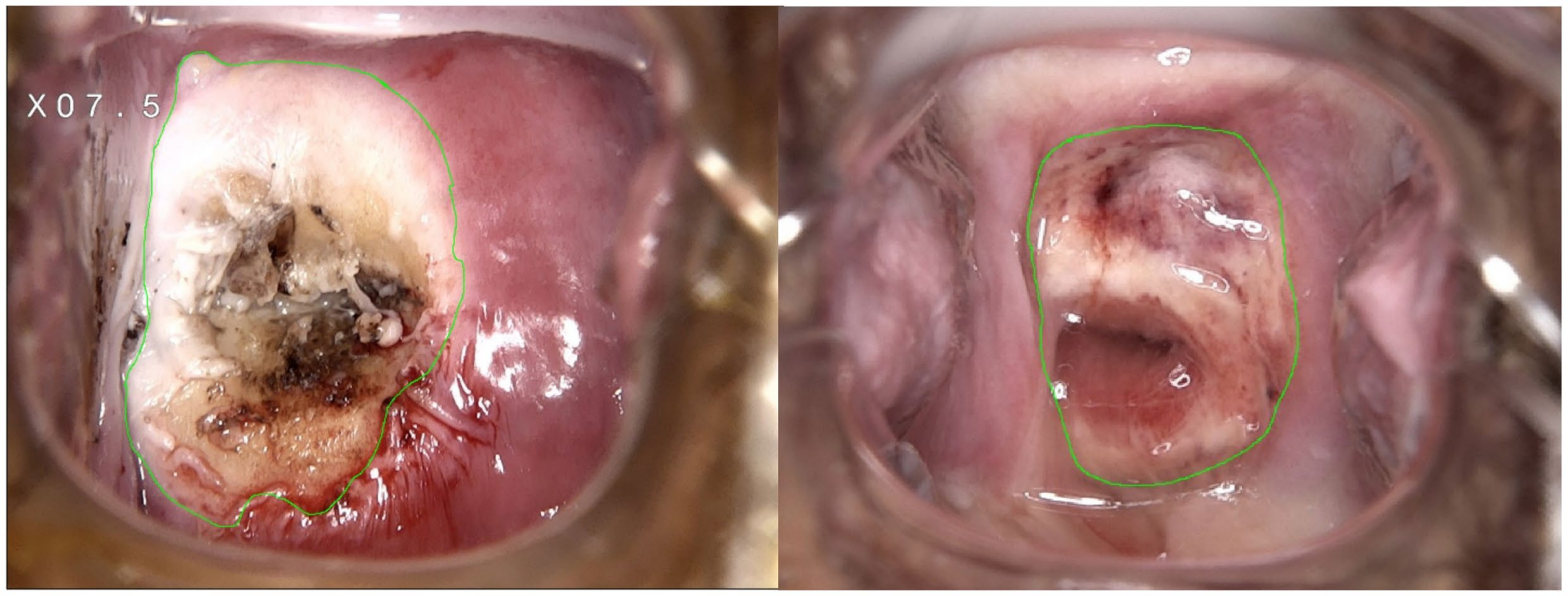
Representative images of wound area measurement with ImageJ software immediately and one month after cervical treatment.

Compared with the control group, the experimental group had a lower wound healing rate 83.77 (62.04, 97.09) % vs. 98.64(97.10, 99.46)%, *p* < 0.001. The Box and whisker plot of the wound healing rate for the two group was shown in Supplementary Figure 1. Also the experimental group had a higher scab non-shedding rate 24.14% (7/29) on the 30th day after treatment when compared with control group (24.14% vs. 3.22%, *p* = 0.024), as shown in Table 2 and Figures 1−4.

**Table 2:** The wound healing status in the experimental group and control group.

Indices	Experimental group (*n* = 29)	Control group (*n* = 31)	*U* or *X*^2^ value	*p*-value
Wound healing rate (%)	83.77(62.04, 97.09)	98.64(97.10, 99.46)	150.00	<0.001
Scab still in place	7 (24.14%)	1 (3.22%)	5.670	0.024
Scab shedding	22 (75.86%)	30 (96.78%)		

The healing rate of cervical wounds in the experimental group was significantly lower than that in the control group 83.77 (62.04, 97.09) % vs 98.64(97.10, 99.46)%, *p* < 0.001, and the cab non-shedding rate on the 30th day after treatment in the experimental group was significantly higher when compared with control group (24.14% vs 3.22%, *p* = 0.024).

We conducted further analysis to explore the potential correlation between delayed wound healing and the timing of SARS-CoV-2 infection in the experimental group. Out of the 7 patients who experienced delayed wound healing, 5 patients (71.43%) contracted SARS-CoV-2 within 2 weeks after undergoing cervical treatment, only 2 (2/7 or 28.57%) patients infected with delayed wound healing infected SARS-CoV-2 2 weeks after cervical treatment. It is worth noting that in the control group, there was only 1 patient who experienced poor wound healing, and the cervical treatment of this patient was conducted 45 days after SARS-CoV-2 infection.

Pre-treatment vaginal discharge tests for all patients exhibited normal levels of white blood cell counts and leukocyte esterase, with no detection of trichomonas vaginalis, pseudohyphae, or budding spores in both pre and post-treatment assessments. We compared the correlation between white blood cell count and leukocyte esterase in vaginal discharge with cervical healing. The results of the chi-square test for contingency table revealed no significant correlation between white blood cell count or leukocyte esterase in vaginal discharge and delayed wound healing of the cervix (defined as the non-shedding of scabs after 1 month of treatment) (*p* = 0.947 and 0.970, respectively), as shown in Tables 3, 4.

**Table 3:** Correlation between leukocyte esterase in vaginal discharge and delayed wound healing following cervical treatment.

Indices	Number of patients (*n*)	Leukocyte esterase testing of vaginal discharge		
		Negative (−)	+	++
Scab shedding	52	18	18	16
Scab still in place	8	3	3	2
*χ*^2^ value	0.110			
*p-*value	0.947			

The correlation between leukocyte esterase of vaginal discharge and delayed wound healing following cervical treatment, the results of the chi-square test for contingency table revealed no significant correlation between leukocyte esterase in vaginal discharge and delayed wound healing after cervical treatment (*p* = 0.947).

**Table 4:** Correlation between white blood cell count of vaginal discharge and delayed wound healing following cervical treatment.

Indices	Number of patients (*n*)	White blood cell count of vaginal discharge			
		0−5/HPF	5−15/HPF	15−30/HPF	>30/HPF
Scab shedding	52	12	6	10	24
Scab still in place	8	2	1	2	3
*χ*^2^ value	0.247				
*p*-value	0.970				

The correlation between white blood cell count of vaginal discharge and delayed wound healing following cervical treatment, the results of the chi-square test for contingency table revealed no significant correlation between white blood cell count of vaginal discharge and delayed wound healing after cervical treatment (*p* = 0.970).

HPF, high-power field.

## 5 Discussion

To the best of our knowledge, this is the first study to explore the impact of SARS-CoV-2 infection on the cervical wound healing after cervical treatment. We found patients who contract SARS-CoV-2 shortly after undergoing cervical treatment, such as loop electrosurgical excision or radiofrequency/focused ultrasound ablation, may experience a notable decrease in the rate of cervical wound healing. These findings suggest that patients who develop SARS-CoV-2 or other illnesses that cause systemic inflammatory responses shortly after cervical treatment may experience delayed healing of cervical wounds.

The healing of wounds is a complex process that involves four main stages: hemostasis, inflammation, cell proliferation, and tissue remodeling (17). Immune dysregulation during wound healing, such as increased local necrotic tissue, poor vascular conditions, high levels of pro-inflammatory cytokines, proteases, reactive oxygen species, and other molecules, as well as infections caused by various pathogens, can result in abnormal immune cell recruitment, imbalanced protein hydrolysis, and impaired vascular formation. These factors can cause the wound to remain in the inflammation stage, leading to delayed healing or chronic wounds (18).

Multiple factors may contribute to delayed healing of cervical wounds in SARS-CoV-2-infected patients undergoing cervical treatment. Firstly, SARS-CoV-2 infection can inhibit interferon signaling pathways, which reduces the expression levels of genes stimulated by interferon type I (IFN-I) and type III (IFN-III) (19). Additionally, it can cause an increase in the expression levels of IL-6 and other inflammatory cytokines, leading to prolonged inflammation during the wound healing process and delayed healing (20, 21). Secondly, SARS-CoV-2 infection can cause hypoxia and vascular dysfunction, further compromising the healing process (22, 23). Lastly, SARS-CoV-2 infection can cause coagulopathy, resulting in excessive bleeding and further delaying the healing process (24, 25).

The white blood cell count in vaginal discharge is an indicator of vaginal inflammation. Additionally, leukocyte esterase can be used to assess inflammatory reactions in the vagina. Through our examination of vaginal discharge, we observed no statistically significant differences in white blood cell count and leukocyte esterase levels between patients with normal wound healing and those with delayed healing following cervical treatment. These findings suggest that delayed wound healing in the cervix is not predominantly attributed to cervical infection, but rather may be associated with compromised immune function and diminished reparative capacity of the body. Last year, Stein et al. (26) conducted a thorough autopsy on 44 deceased patients who had been infected with SARS-CoV-2. Aside from the respiratory tract, the virus’s RNA was found in tissues including the thyroid, esophagus, spleen, appendix, adrenal gland, ovaries, testes (including mature sperm), and endometrium. Currently, there is no conclusive evidence to suggest that SARS-CoV-2 RNA is present in vaginal or cervical secretions. In this study, SARS-CoV-2 antigen tests were performed on vaginal and cervical secretions extracted from patients with poor wound healing, yielding negative results. Therefore, it is speculated that the increased risk of poor wound healing in SARS-CoV-2-infected patients is not directly related to the virus but rather due to systemic immune damage, inflammatory reactions, and hypoxia.

Similar to the highly pathogenic severe acute respiratory syndrome coronavirus (SARS-CoV) in 2003 and the Middle East respiratory syndrome coronavirus (MERS-CoV) in 2012, SARS-CoV-2 infection can also trigger cytokine storm syndrome (CSS) (27, 28). CSS is a crucial mechanism contributing to poor wound healing in SARS-CoV-2 patients. Infection after cervical treatment can cause acute symptoms that trigger CSS, leading to excessive immune response in the body. This can result in continuous activation and proliferation of lymphocytes and macrophages, secretion of large amounts of cytokines, and abnormal wound hemostasis, inflammation, proliferation, and tissue remodeling processes. Many studies have found that SARS-CoV-2 infection rapidly activates inflammatory T lymphocytes and monocytes, producing more granulocyte-macrophage colony-stimulating factor, IL-6, IL-10, and other serum inflammatory cytokines, while decreasing peripheral blood lymphocyte counts. This provides an important theoretical basis for exploring the poor wound healing in SARS-CoV-2-infected patients (29).

The pulmonary pathology associated with SARS-CoV-2 infection primarily involves diffuse alveolar damage, deep airway injury, and pulmonary consolidation, with thick “mucus plugs” that can obstruct the lower respiratory tract (30). As a result, Patients infected with SARS-CoV-2 exhibit varying degrees of hypoxia. Prolonged hypoxia has been found to reduce collagen matrix generation, delay the formation of granulation tissue, and impede wound healing. Thus, the systemic tissue hypoxia in SARS-CoV-2 patients may hinder wound healing at various stages, from local inflammation to tissue remodeling. Delayed wound healing after cervical treatment may lead to scar formation and potential cervical dysfunction. Recent reports have indicated a rising number of re-infections or multiple SARS-CoV-2 infections in individuals. To minimize the adverse consequences of delayed cervical wound healing and avoid any negative impact on scar healing, it is recommended that patients who have undergone cervical treatment avoid contracting SARS-CoV-2 within the first month following the procedure.

### Limitations

This study has the following limitations. Firstly, although delayed cervical wound healing was found in patients who infected SARS-CoV-2 shortly after receiving cervical treatment, the inflammatory biomarkers and immunological indices was not routine conducted for the patients, and all patients infected with SARS-CoV-2 had mild symptoms, so it is unclear whether the mechanism is related to systemic inflammation. Secondly, the time frame for patients included in this study who were infected with SARS-CoV-2 after receiving cervical treatment was set within one month after the procedure, and different infection times may have impact on cervical wound healing. Thirdly, the colposcopy is usually scheduled one month after treatment, so we cannot determine the recovery status of cervical wounds in patients at 15 days, 1.5 months, or even 2 months after treatment. However, we did find that the wound healing time of cervical wounds was significantly prolonged in patients who contracted COVID-19 shortly after cervical treatment. Furthermore, this study is a retrospective analysis conducted during a specific period of the SARS-CoV-2 pandemic, with a small sample size and the inability to carry out prospective randomized controlled trials. Nevertheless, the findings have already demonstrated that SARS-CoV-2 infection can affect the wound healing rate of cervical treatments.

## Conclusion

In this retrospective cohort study with a small sample size, we observed a higher incidence of poor wound healing in SIL patients who contracted SARS-CoV-2 during the wound healing stage of cervical treatment. Therefore, it is recommended for patients to avoid contracting viruses such as SARS-CoV-2 in the first month after cervical treatment to reduce the risk of delayed healing and minimize adverse outcomes associated with scar healing.

## Data availability statement

The original contributions presented in the study are included in the article/Supplementary material, further inquiries can be directed to the corresponding authors.

## Ethics statement

The studies involving humans were approved by the Ethics Committee of Guangxi Zhuang Autonomous Region People's Hospital. The studies were conducted in accordance with the local legislation and institutional requirements. The participants provided their written informed consent to participate in this study.

## Author contributions

LX: conceptualization, methodology, and draft writing. YW and CL: writing and editing. RZ and ZW: supervision. All authors have read and agreed to the published version of the manuscript. All authors contributed to the article and approved the submitted version.
