# Supplementary figures and images for: The impact of Sars-Cov-2 infection on the wound healing of cervical treatment in patients with squamous intraepithelial lesions: a retrospective cohort study
Source: Front Med (Lausanne). 2023 Dec 7;10:1222767. doi: 10.3389/fmed.2023.1222767 (PMC10733498; doi:10.3389/fmed.2023.1222767)

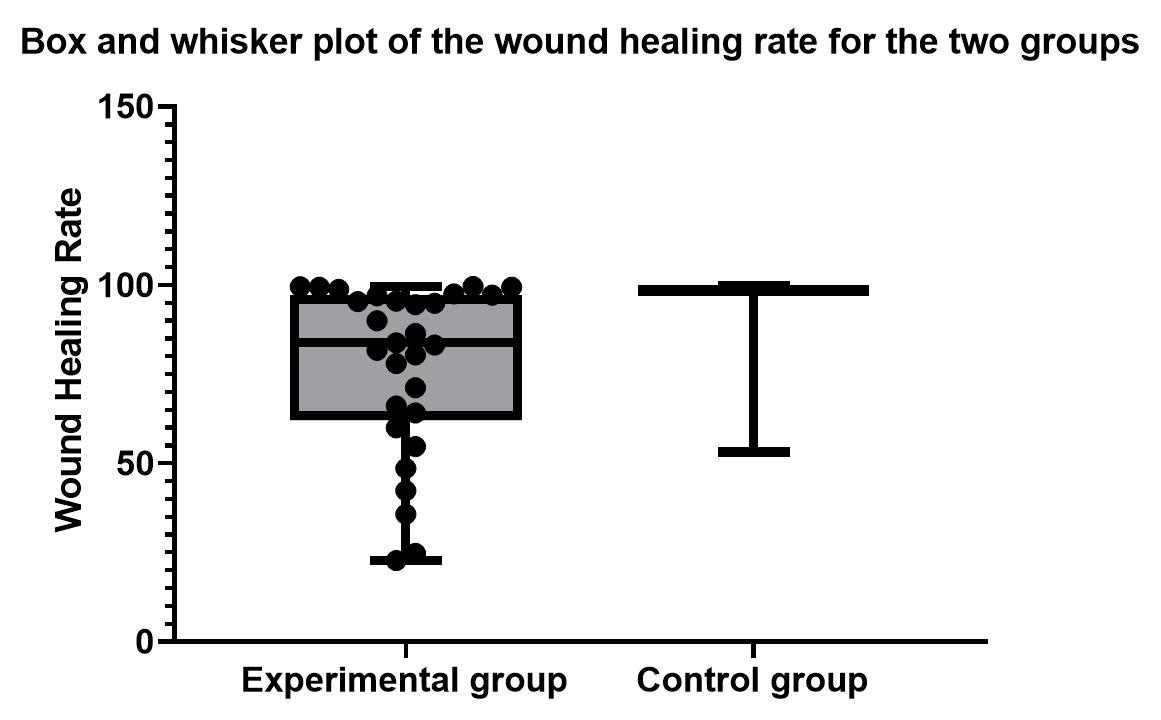

Supplement: Supplementary file 1 [file Image_1.JPEG]
